# Response: Commentary: How to Make the Ghosts in my Bedroom Disappear? Focused-Attention Meditation Combined with Muscle Relaxation (MR Therapy)—A Direct Treatment Intervention for Sleep Paralysis

**DOI:** 10.3389/fpsyg.2017.00760

**Published:** 2017-05-16

**Authors:** Baland Jalal

**Affiliations:** Behavioural and Clinical Neuroscience Institute and Department of Psychiatry, University of CambridgeCambridge, UK

**Keywords:** sleep paralysis, treatment, REM sleep, CBT interventions, intervention

There are currently few treatments available for sleep paralysis (SP), as noted in my article. Nonetheless as I also explain, there have been some initial attempts to treat SP (e.g., Ohaeri et al., 1992; Stores, 1998). My colleague Devon Hinton is one of the first to have carried out work in treating SP among traumatized Cambodians using the application of cognitive-behavioral therapy (CBT) techniques (an approach called Culturally Adapted CBT or CA-CBT); this work focuses on psycho-education and modifying catastrophic cognitions about the attack (see Hinton et al., 2005a,b). Hinton and I have recently extended this work using CA-CBT to treat SP among traumatized Egyptians and South Africans (Jalal et al., 2017, in review). Sharpless and Doghramji (2015) similarly provides a CBT approach (CBT-ISP) to treating SP, cited in my article. This approach seems to have been inspired by the early literature on the application of CBT in treating SP and the work of Hinton in particular, as the authors themselves note, “the successful application of CBT techniques and principals can be found in the literature, and these were important in the creation of our own approach. The most extensive of these are found in the work of Devon Hinton” (p. 238). While there are similarities between these CBT-type interventions, and Meditation-Relaxation (MR) Therapy for SP (Jalal, 2016), for instance, derived from standard CBT theory and panic disorder models, there are some very fundamental differences.

MR therapy is derived from my empirical work on SP in North America, Scandinavia, Southern Europe, and the Middle East (e.g., Jalal and Hinton, 2013, 2015, 2016; Jalal et al., 2014a,b, 2015); as well as my neuroscientific accounts for SP hallucinations with my colleague, Vilayanur S. Ramachandran (Jalal and Ramachandran, 2014, 2017). This empirical and theoretical work run through the heart of MR Therapy, and has resulted in a novel model of SP occurrence and frequency, worsening of somatic symptoms, panic, and hallucinations (i.e., of ghost-like figures)—the so-called, Panic-Hallucination Model (e.g., Jalal, 2016, p. 2). The quintessential goal of MR therapy is to provide a complete and systematic step-by-step method to deal with SP directly during the attack, using a very specific type of meditation (inward focused-attention meditation) combined with muscle relaxation; rather than recommending “mindfulness” or “self-soothing talk,” for instance, as potentially beneficial add-on elements. Unlike other treatments, while MR Therapy encourages psycho-education and rehearsal of SP that are applied outside episodes [the former used by Hinton and others (see above), and the latter to my knowledge first proposed by Sharpless and Doghramji, 2015], these are secondary to the treatment itself (i.e., not part of the treatment *per se* but optional elements). As opposed to stressing cognitive reappraisals outside SP, MR Therapy emphasizes reappraisals and emotional distancing during SP. Similarly, and crucially, MR Therapy does not favor disrupting SP episodes, but instead the use of specific meditation-relaxation techniques to cope with the event as it eventually passes on its own. Unlike other approaches, MR Therapy discourages: (1) movement during SP; i.e., given the lack of sensory feedback from the body (deafferentation), which could lead to pain and spasms in limbs (see also Cheyne et al., 1999), and possibly trigger hallucinations (e.g., Jalal and Ramachandran, 2014, 2017); (2) controlling breathing (e.g., using deep breathing techniques), given REM-respiration features (e.g., shallow rapid breathing) which could result in chest pain; (3) focusing on somatic symptoms that are usually unpleasant; as opposed to engaging in inward-attention meditation and focusing attention *away* from somatic sensations—a vital component of MR Therapy (Jalal, 2016, p. 2–3).

To my reading the CBT-ISP approach best qualifies as a comprehensive CBT manual, the first of its kind, mainly emphasizing the treatment of SP outside of episodes (psycho-education, sleep diaries skills training, and cognitive re-interpretations and reappraisals outside SP etc.)—although it certainly also provides recommendations on how to deal with SP during the attack (deep breathing and wiggling a toe etc.). MR Therapy was specifically designed to give a complete and systematic step-by-step account on how to deal with SP *directly* during the actual attack—the very crux of this approach—based on the aforementioned empirical and theoretical work. In other words, MR Therapy is exclusively a *direct* treatment; and, indeed, any techniques applied outside of SP (psychoeducation, imaginary rehearsal, etc.) are not part of the intervention itself, but may still be useful as ways to augment the potential treatment effects. Nonetheless, whether MR Therapy—technically speaking—“qualifies” as the “first *direct* treatment for sleep paralysis,” with emphasis on “direct” here (i.e., given its key focus on dealing with SP directly during the attack in a systematic fashion) is a matter of interpretation and arguably of definition. Leaving room for such nuance and subjectivity in opinion (lest I should inadvertently step on someone's toes), I qualified in my article (Jalal, 2016, p. 2), that indeed “to the best of my knowledge, there are no direct treatment interventions available for SP….” However, frankly, whether MR Therapy is considered the first, second, or fifth in line, doesn't concern me all that much—there is no Nobel prize at stake here. What ultimately matters is how such interventions can have real-world practical value for SP sufferers around the world.

## Author contributions

The author confirms being the sole contributor of this work and approved it for publication.

### Conflict of interest statement

The author declares that the research was conducted in the absence of any commercial or financial relationships that could be construed as a potential conflict of interest.
